# Primary renal lymphoma mimicking renal cell carcinoma

**DOI:** 10.4103/0970-1591.70591

**Published:** 2010

**Authors:** Sanju Cyriac, Rajendranath Rejiv, Sundersingh Shirley, Gnana T. Sagar

**Affiliations:** Department of Medical Oncology, Cancer Institute (WIA), Chennai, India; 1 Department of Pathology, Cancer Institute (WIA), Chennai, India

**Keywords:** Imaging, primary renal lymphoma, renal cell carcinoma

## Abstract

Primary renal lymphoma is very rare. Lymphomatous involvement of the kidney is often seen as a part of disseminated disease. The prognosis is usually poor with median survival less than a year. It is essential to differentiate between renal cell carcinoma and renal lymphoma in patients presenting with solitary renal masses. We present a 52-year-old lady who presented with a solitary renal mass and was diagnosed to have primary lymphoma of the kidney and discuss briefly about primary renal lymphoma.

## INTRODUCTION

Kidney is a common site of involvement for extranodal lymphomas.[1] Majority of them are diagnosed postmortem and often seen as part of a disseminated disease. Primary Renal lymphomas (PRL) are defined as lymphomas arising in the renal parenchyma and not invasion from an adjacent lymphomatous mass. Clinico-radiologically it may mimic renal cell carcinoma.[2] The prognosis is very poor with median survival less than a year. PRL is often considered a systemic disease manifesting initially in the kidneys. Imaging plays an important role in PRL. The most common feature is that of multiple nodular masses. Preoperative biopsy is worthwhile in patients with atypical radiological features, since it may avoid nephrectomy. The most common histological subtype encountered is diffuse large B cell lymphoma (DLBCL). The treatment of non-Hodgkin’s Lymphoma (NHL) has been revolutionized with addition of Rituximab to the standard chemotherapy. This may overcome the poor outlook of this rare presentation of lymphoma.

## CASE REPORT

A 52-year-old woman presented with flank pain and intermittent hematuria of six months duration, without any fever or weight loss. She had a vague mass palpable in the left hypochondrium. Peripheral lymph nodes were not palpable.

**Figure 1:**
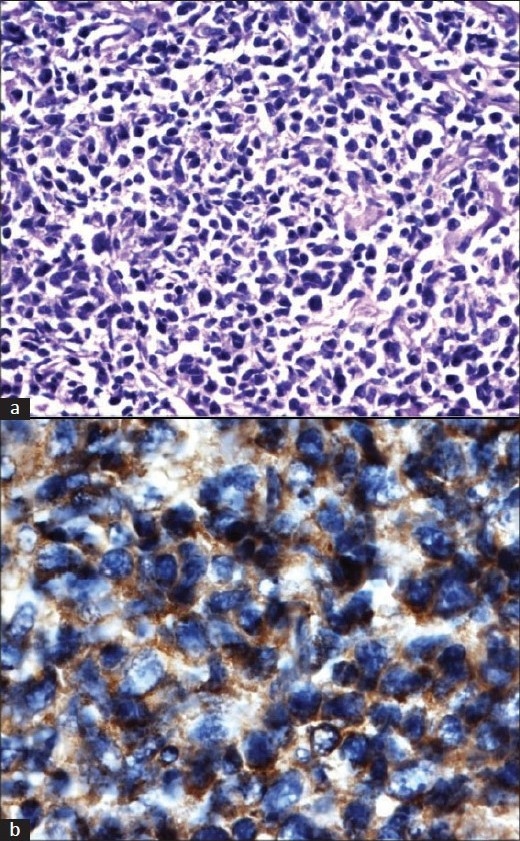
(a) Histopathology of trucut biopsy of renal mass demonstrating diffuse growth of round cells with moderate cytoplasm and prominent nucleoli. Increased mitotic figures seen (b) CD 20 positivity

Complete blood picture and renal/liver function tests were normal. Computerized tomography (CT) abdomen revealed a large (12 × 11.5 cm) solid, well encapsulated, homogenously and mildly enhancing mass occupying mid and lower pole regions of right kidney. There was a large cyst demonstrable in the left kidney. [Figure 1] Pre and paracaval lymph nodes were also demonstrable encasing the right renal vessels but with preserved flow.

**Figure 2:**
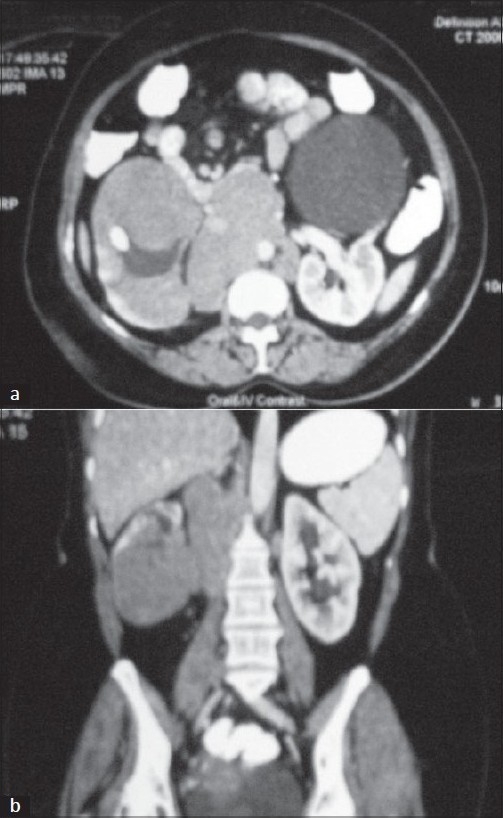
(a) A mass in the right kidney in the lower pole with adjacent lymphadenopathy, encasing the vessels. A large cyst is demonstrable in the left kidney. (b) Coronal view of the mass and the adjacent adenopathy

CT guided trucut biopsy was done. It was suggestive of Non Hodgkins Lymphoma (NHL). [Figure 2] Immunohistochemistry was performed. LCA, CD20, CD79a, BCL-2 were positive. Ki67 was 40 – 50 %, Cyclin D1 and Tdt were negative. She was diagnosed as NHL, DLBCL. Bilateral bone marrow studies were normal. She was initiated as CHOP chemotherapy and is responding to treatment.

## DISCUSSION

Renal involvement in NHL is usually seen as a part of disseminated disease. The reported incidence is as high as 47% in autopsy series of lymphomas but clinically recognized in only up to 15% of patients.[1] Primary renal NHL is defined as a NHL arising in the renal parenchyma and not invasion from an adjacent lymphomatous mass.[1] PRL usually affects adults. The clinical presentations include flank pain, hematuria, abdominal mass, fever and weight loss. Acute renal failure is yet another clinical presentation described in literature.

Kidney is not a lymphoid organ. Hence the very existence of lymphomas of kidney was questioned by some investigators. The proposed pathogenetic mechanisms include: origin in the subcapsular lymphatics, seeding via hematogenous route, an extension from retro peritoneal disease or inflammatory disease with a lymphoplasmacytic infiltrate.[2]

The role of imaging is very crucial. The most commonly encountered pattern is that of multiple soft tissue masses, ranging from 1-3 cms, with minimal enhancement after contrast compared to surrounding renal parenchyma.[3] Solitary lesion, as seen in our patient, makes it a difficult diagnosis as it resembles the more common renal cell carcinoma (RCC). The differentiating features include absence of calcification, post contrast homogenous attenuation, absence of renal vein thrombus and absence of a mass effect on renal vessels and pelvicalyceal system in PRL. However, this situation still demands a biopsy to rule out RCC. Other less commonly seen patterns include, enlarged non enhancing kidneys, direct invasion of renal sinus and hilum by bulky retroperitoneal mass or a diffuse perirenal infiltration encasing the kidney.[4] Most of the patients also have adjacent retroperitoneal adenopathy. MRI is currently becoming the imaging modality of choice for evaluation of renal lesions. Lower signal intensity on unenhanced T1-weighted images than normal renal cortex and less enhancement on early gadolinium-enhanced images differentiates Renal Lymphoma from RCC.

Diffuse Large B Cell Lymphoma (DLBCL) is the most common histology though encountering a follicular lymphoma, small lymphocytic lymphoma or MALToma is not unusual.

The prognosis is reported poor universally. Median survival is less than a year.[5] PRL is considered as a systemic disease, presenting with renal manifestation. However, nephrectomy can be avoided if a preoperative diagnosis is made. Patients with atypical features of RCC, therefore, should undergo a preoperative percutaneous renal biopsy. The sensitivity and specificity of renal biopsy are 70% to 92% and 100%, respectively, with accuracy close to 90%.[5] The treatment of renal lymphoma depends on the primary histological subtype. The addition of Rituximab to the standard CHOP chemotherapy may improve the dismal outcome reported so far.

Our patient presented with a solitary mass lesion in the right kidney. Though lymph nodes were demonstrable in the retroperitoneum, the absence of disease elsewhere points towards kidney as the probable site of origin. This case is important to the practicing oncologists to get enlightened about primary renal lymphoma which can radiologically and clinically mimic renal cell carcinoma. Making a preoperative diagnosis can avoid unnecessary nephrectomies in such cases.
